# Individual patient data to allow a more elaborated comparison of trial results with real-world outcomes from first-line immunotherapy in NSCLC

**DOI:** 10.1186/s12874-022-01760-0

**Published:** 2023-01-03

**Authors:** R. K. Ismail, F. M. N. H. Schramel, M. van Dartel, A. M. G. Pasmooij, C. M. Cramer-van der Welle, D. L. Hilarius, A. de Boer, M. W. J. M. Wouters, E. M. W. van de Garde

**Affiliations:** 1 Dutch Institute for Clinical Auditing, Leiden, the Netherlands; 2 Division of Pharmacoepidemiology and Clinical Pharmacology, Utrecht Institute for Pharmaceutical Sciences, Utrecht, The Netherlands; 3 Medicines Evaluation Board, Utrecht, The Netherlands; 4 Department of Pulmonary Diseases, St Antonius Hospital, Nieuwegein, The Netherlands; 5 Santeon Hospital Group, Utrecht, The Netherlands; 6 Department of Clinical Pharmacy, Rode Kruis Hospital, Beverwijk, The Netherlands; 7 Department of Biomedical Data Sciences, Leiden University Medical Center, Leiden, The Netherlands; 8 Department of Clinical Pharmacy, St Antonius Hospital, Nieuwegein, Utrecht, The Netherlands

**Keywords:** NSCLC, Nivolumab, Real-world, Efficacy- effectiveness gap

## Abstract

**Background:**

Many studies have compared real-world clinical outcomes of immunotherapy in patients with metastatic non-small cell lung cancer (NSCLC) with reported outcomes data from pivotal trials. However, any differences observed could be only limitedly explored further for causation because of the unavailability of individual patient data (IPD) from trial participants. The present study aims to explore the additional benefit of comparison with IPD.

**Methods:**

This study compares progression free survival (PFS) and overall survival (OS) of metastatic NSCLC patients treated with second line nivolumab in real-world clinical practice (*n* = 141) with IPD from participants in the Checkmate-057 clinical trial (*n* = 292). Univariate and multivariate Cox proportional hazards models were used to construct HRs for real-world practice versus clinical trial.

**Results:**

Real-world patients were older (64 vs. 61 years), had more often ECOG PS ≥ 2 (5 vs. 0%) and were less often treated with subsequent anti-cancer treatment (28.4 vs. 42.5%) compared to trial patients. The median PFS in real-world patients was longer (3.84 (95%CI: 3.19-5.49) vs 2.30 (2.20-3.50) months) and the OS shorter than in trial participants (8.25 (6.93-13.2) vs. 12.2 (9.90-15.1) months). Adjustment with available patient characteristics, led to a shift in the hazard ratio (HR) for OS, but not for PFS (HRs from 1.13 (0.88-1.44) to 1.07 (0.83-1.38), and from 0.82 (0.66-1.03) to 0.79 (0.63-1.00), respectively).

**Conclusions:**

This study is an example how IPD from both real-world and trial patients can be applied to search for factors that could explain an efficacy-effectiveness gap. Making IPD from clinical trials available to the international research community allows this.

**Supplementary Information:**

The online version contains supplementary material available at 10.1186/s12874-022-01760-0.

## Introduction

The treatment landscape of metastatic lung cancer patients has changed over recent years [1]. Chemotherapy used to be the cornerstone therapy for metastatic non-small cell lung cancer (NSCLC) patients, but the introduction of immunotherapy has positively changed the clinical outcomes of these patients [2–4]. Immunotherapy is increasingly more prescribed in the Netherlands. The Dutch Lung Cancer Audit showed that immunotherapy-based treatments consisted of 15% of all treatments in 2015 and increased to 57% in 2019 [5].

The phase-III marketing authorization trials researching immunotherapy in NSCLC patients used strict in- and exclusion criteria [4, 6, 7]. Patients treated in real-world practice can differ from these trial patients, leading to different clinical outcomes, also known as the efficacy-effectiveness (EE) gap [8]. Because of the unavailability of individual patient data (IPD) from clinical trials, a common approach for comparing trial and real-world patients is using Kaplan-Meier curves from scientific publications. These are digitized with software, such as DigitizeIt, to allow comparison between trial and real-world patients and to measure the hazard ratio (HR) between the curves [8, 9]. Previous Dutch research on immunotherapy treatment (nivolumab and pembrolizumab) also used this approach and showed differences in clinical outcomes between real-world metastatic NSCLC and trial patients [10]. However, further search for causation, for example, through multivariable regression modeling, was not put forward because of unavailable IPD from the respective trials.

Recently, for one of the pivotal trials involved in the Dutch EE gap study, the IPD have come available. The aim of the present study is to explore if individual patient data (IPD) could be helpful to identify factors that explain divergence between outcomes from the nivolumab treatment arm of the Checkmate-057 clinical trial and patients with NSCLC treated in real-world clinical practice.

## Methods

### Data sources

This exploratory study is an in-depth study of the study of Cramer-van der Welle et al [10]*.* The data from that study were re-used. The trial data from the Checkmate-057 trial were collected from the internal ICI database of the Medicines Evaluation Board database.

### Patients and outcomes

The population under study consisted of metastatic nonsquamous NSCLC patients treated with second line nivolumab after prior platinum-containing chemotherapy. Real-world patients were treated with nivolumab in the years 2015 to 2018. Participants in the Checkmate-057 clinical trial were treated before marketing authorization [4]. The outcomes in this study were progression-free survival (PFS) and overall survival (OS).

### Statistical analyses

Patient- and tumor characteristics of the study population were analyzed using descriptive statistics. These included age, gender, stage, Eastern Cooperative Oncology Group Performance Score (ECOG PS), the presence of brain metastases at diagnosis, tumor histology, and programmed death-ligand 1 (PD-L1) expression. Age was categorized in < 70 and ≥ 70 years, since NSCLC has a median onset at age 70 years [11].

The Kaplan-Meier method with log-rank test was used to compare the PFS and OS between real-world and trial patients. Survival times were calculated from the start of nivolumab treatment (real-world patients) or randomization date (trial patients). Patients not reaching the endpoint at data cut-off were censored at the last known alive date. Median follow-up duration was calculated for the study population using the reverse Kaplan-Meier method [12].

Next, analogous to identification of potential confounders, relative changes in the HR were used to identify factors that could explain the difference between real-world and trial patient outcomes. To do so, univariable and multivariable Cox proportional hazards models were used to construct HRs for real-world practice versus clinical trial patients for both outcomes. All patient- and tumor characteristics (see above) were assessed as potential explanatory factors. Theoretically, variables that result in adjustment of the HR towards 1.00 were considered as potential causative for the EE-gap. Since this study does not compare two different treatments but two groups treated similarly, we argue that the influence of long-term survivors on the proportionality of the Cox model is limited. Statistical analyses were stated significant if the *p*-value was < 0.05.

Data handling and statistical analyses were performed using the R software system for statistical computing [13] (version 4.1.0.; packages tidyverse, lubridate, tableone, ggplot2, survival, survminer, gtsummary, forestmodel).

### Ethical statement

The Santeon Institutional Review Board reviewed and approved the original study and the need for informed consent was waived (SDB219-008). For this secondary analysis, all clinical information was provided anonymously.

## Results

### Patient characteristics

A total of 292 metastatic NSCLC patients were treated with nivolumab in the Checkmate-057 trial and 141 patients in real-world clinical practice. The median follow-up time of the real-world and trial patients was respectively 25.2 (95%CI 22.7-32.6) and 18.6 (95%CI 17.6-20.1) months. Real-world patients were older (64 (44-80) years vs 61 (37-84), *p* = 0.003) compared to trial patients. Five percent (*n* = 7) of the real-world patients had an ECOG PS of 2, compared to 0% in trial patients. The trial patients were more often treated with subsequent anti-cancer treatment compared to real-world patients (42.5% vs. 28.4%, *p* = 0.006). These characteristics are presented in Table 1.

**Table 1:** Patient characteristics of metastatic non-small cell lung cancer (NSCLC) patients treated with nivolumab in the randomized controlled trial (RCT) and real-world

	Trial patients	Real-world patients	P-value
n	292	141	
Age at diagnosis in years; median (range)	61 (37-84)	64 (44-80)	0.003
Age in categories; n (%)			0.182
< 70 years	241 (82.5)	108 (76.6)	
≥ 70 years	51 (71.5)	33 (23.4)	
Gender; n (%)			0.962
Male	151 (51.7)	74 (52.5)	
Female	141 (48.3)	67 (47.5)	
Stage; n (%)			0.003
IIIB	20 (6.8)	0	
IV	272 (93.2)	141 (100)	
ECOG PS; n (%)			< 0.001
0	84 (28.8)	44 (31.2)	
1	208 (71.2)	90 (63.8)	
2	0	7 (5.0)	
Presence of brain metastases; n (%)			0.114
Yes	34 (11.6)	25 (17.7)	
No	258 (88.4)	116 (82.3)	
Histology tumor; n (%)			0.006
Adenocarcinoma	270 (92.5)	130 (92.2)	
Large cell carcinoma	7 (2.4)	8 (5.7)	
Adenosquamous	3 (1.0)	0	
Other	11 (3.8)	0	
Not otherwise specified (NOS)	0	3 (2.1)	
PD-L1 expression; n (%)			< 0.001
< 1%	108 (37.0)	33 (23.4)	
1-49%	57 (19.5)	29 (20.6)	
> 50%	66 (22.6)	10 (7.1)	
Unknown	61 (20.9)	69 (48.9)	
Subsequent systemic therapy; n (%)			0.006
Yes	124 (42.5)	40 (28.4)	
No	168 (57.5)	101 (71.6)	

ECOG PS = Eastern Cooperative Oncology Group Performance Score, PD-L1 = Programmed death-ligand 1, RCT = randomized controlled trial.

### Progression-free survival

**Fig. 1:**
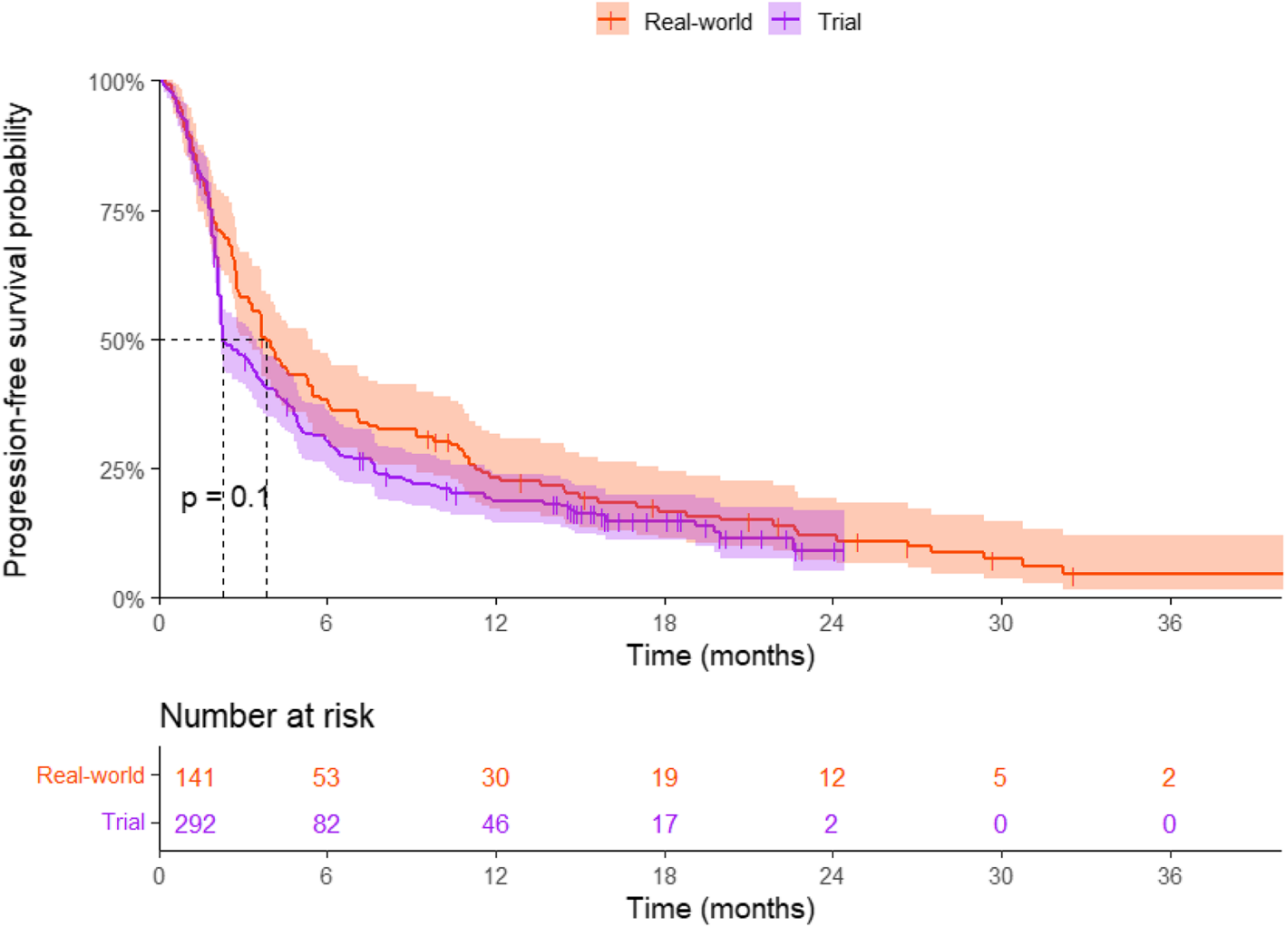
Kaplan-Meier estimate of the progression-free survival (PFS) of metastatic NSCLC patients treated with nivolumab in the clinical trial versus real-world. The progression-free survival time was calculated from randomization date to first progression in clinical trial patients and from start of nivolumab treatment to first progression in real-world patients

**Fig. 2:**
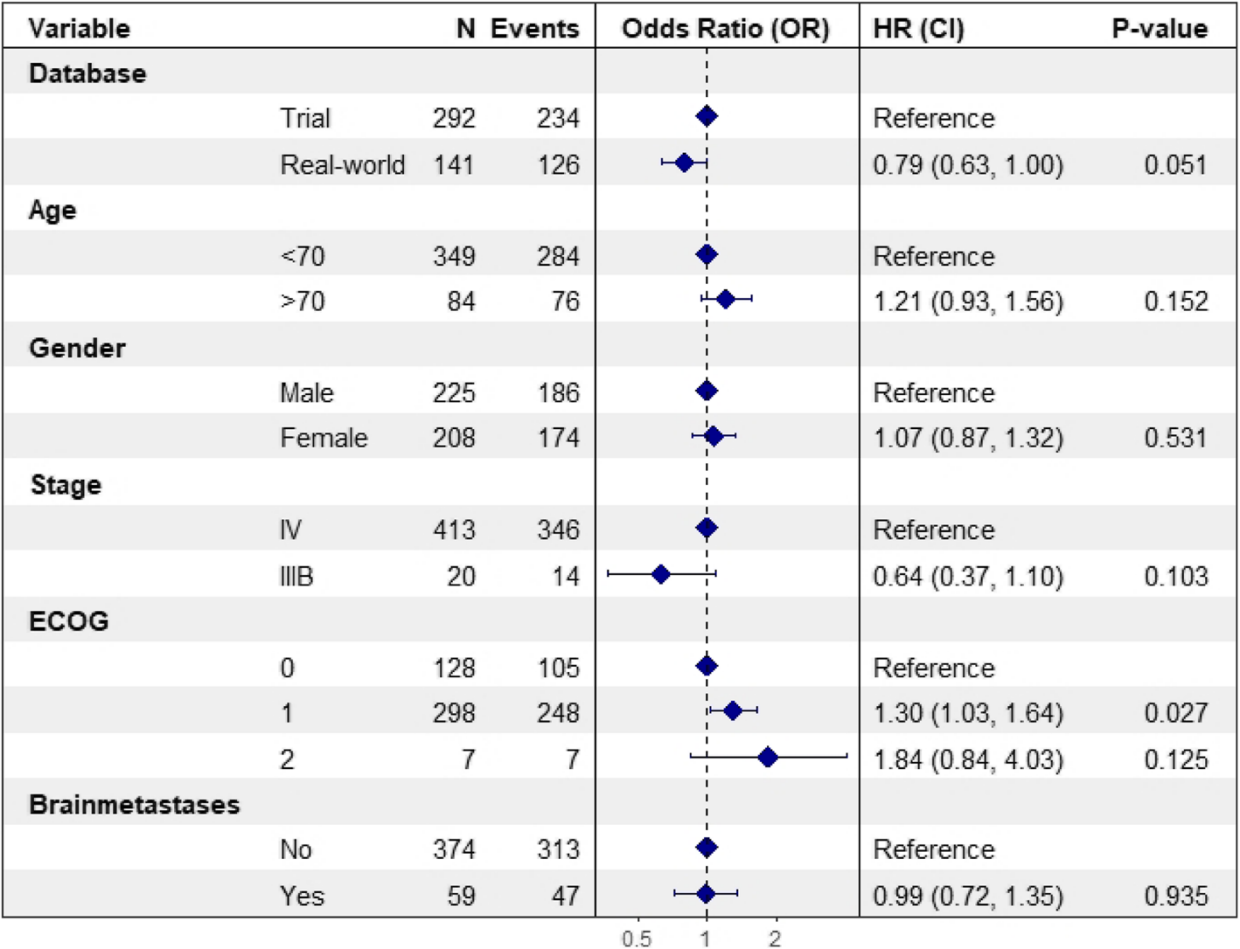
Forest plot visualizing multivariate proportional hazard cox regression model of factors associated with the progression-free survival (PFS) of metastatic NSCLC patients

The median PFS of real-world patients was 3.84 (95%CI: 3.19-5.49) months compared to 2.30 (95%CI: 2.20-3.50) months in trial patients (*p* = 0.104) (Fig. 1). The unadjusted HR for real-world versus trial was 0.82 (95%CI: 0.66-1.03). Patient characteristics associated with PFS were ECOG PS 1 (*p* = 0.018) and PD-L1 expression > 50% (*p* < 0.001) (Table 2). The multivariate Cox model, including all patient characteristics, yielded an adjusted HR for real-world versus trials of 0.79 (0.63-1.00) (Fig. 2).

**Table 2:** Univariate analysis (PFS) of the pooled dataset including real-world and clinical trial patients

Variable	n	OR (95%CI)	P-value
Population			
Trial patients	292	Reference	
Real-world patients	141	0.82 (0.66-1.03)	0.088
Age (years)			
< 70	349	Reference	
≥ 70	84	1.21 (0.94-1.56)	0.146
Gender			
Male	225	Reference	
Female	208	1.02 (0.83-1.25)	0.852
Stage			
IV	413	Reference	
IIIB	20	0.72 (0.42-1.22)	0.221
ECOG PS			
0	128	Reference	
1	298	1.32 (1.05-1.66)	0.018
2	7	1.68 (0.78-3.62)	0.182
Presence of brain metastases			
No	374	Reference	
Yes	59	0.95 (0.7-1.29)	0.749
Histology tumor			
Adenocarcinoma	400	Reference	
Large cell carcinoma	15	1.18 (0.68-2.06)	0.558
Adenosquamous	3	1.15 (0.37-3.59)	0.808
Other	11	0.97 (0.48-1.97)	0.943
Not otherwise specified (NOS)	3	1.74 (0.56-5.44)	0.339
PD-L1 expression			
< 1%	141	Reference	
1-49%	86	0.85 (0.63-1.14)	0.272
> 50%	76	0.53 (0.39-0.74)	< 0.001
Unknown	130	0.93 (0.72-1.21)	0.601

*ECOG PS* Eastern Cooperative Oncology Group Performance Score, *PD-L1* Programmed death-ligand 1, *OR* Odds Ratio

CI = Confidence Interval , ECOG = Eastern Cooperative Oncology Group Performance Score, OR = Odds Ratio. Analysis from the pooled dataset including real-world and clinical trial patients.

### Overall survival

**Fig. 3:**
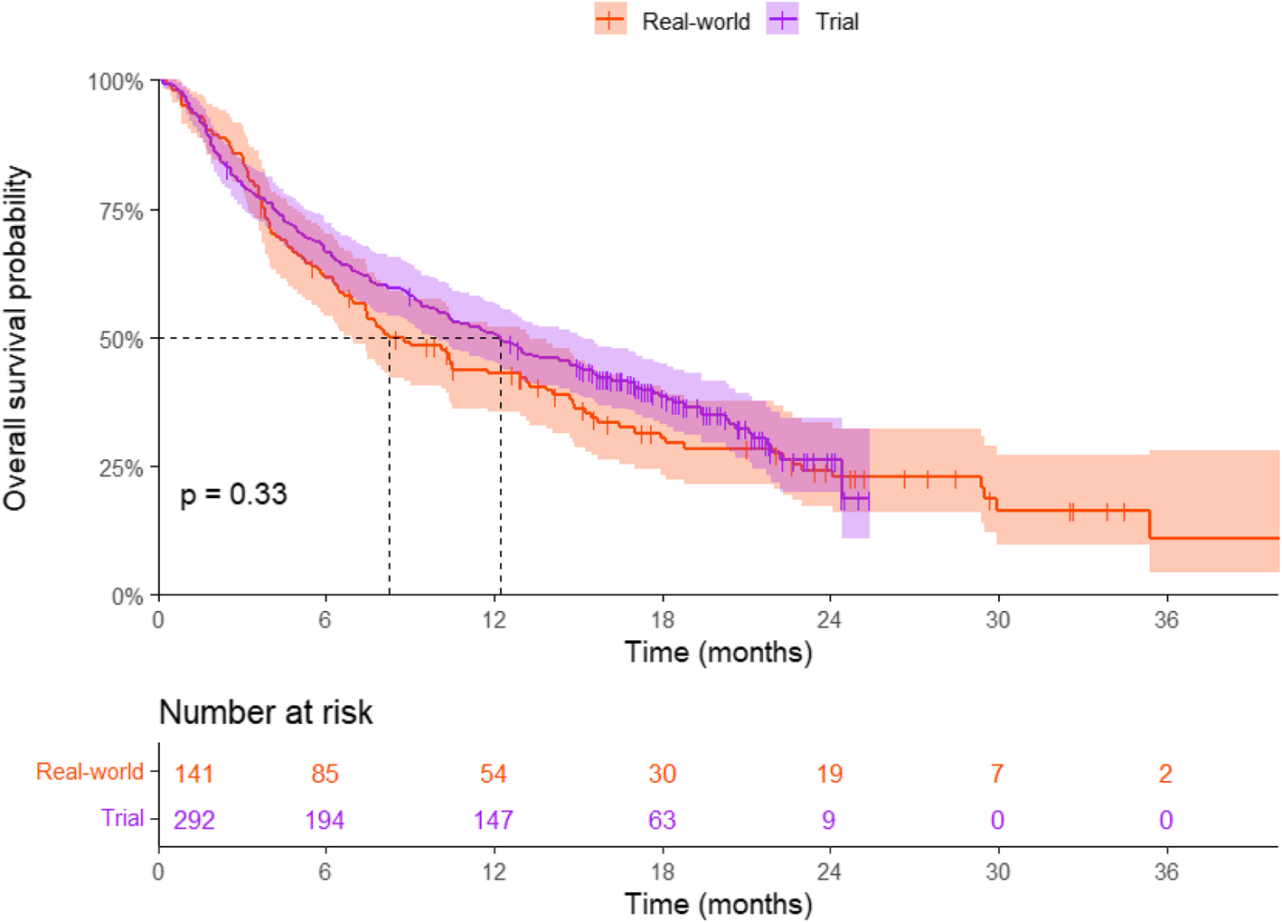
Kaplan-Meier estimate of the overall survival (OS) of metastatic NSCLC patients treated with nivolumab in the clinical trial versus real-world. The overall survival time was calculated from randomization date to death in clinical trial patients and from start of nivolumab treatment to death in real-world patients

**Fig. 4:**
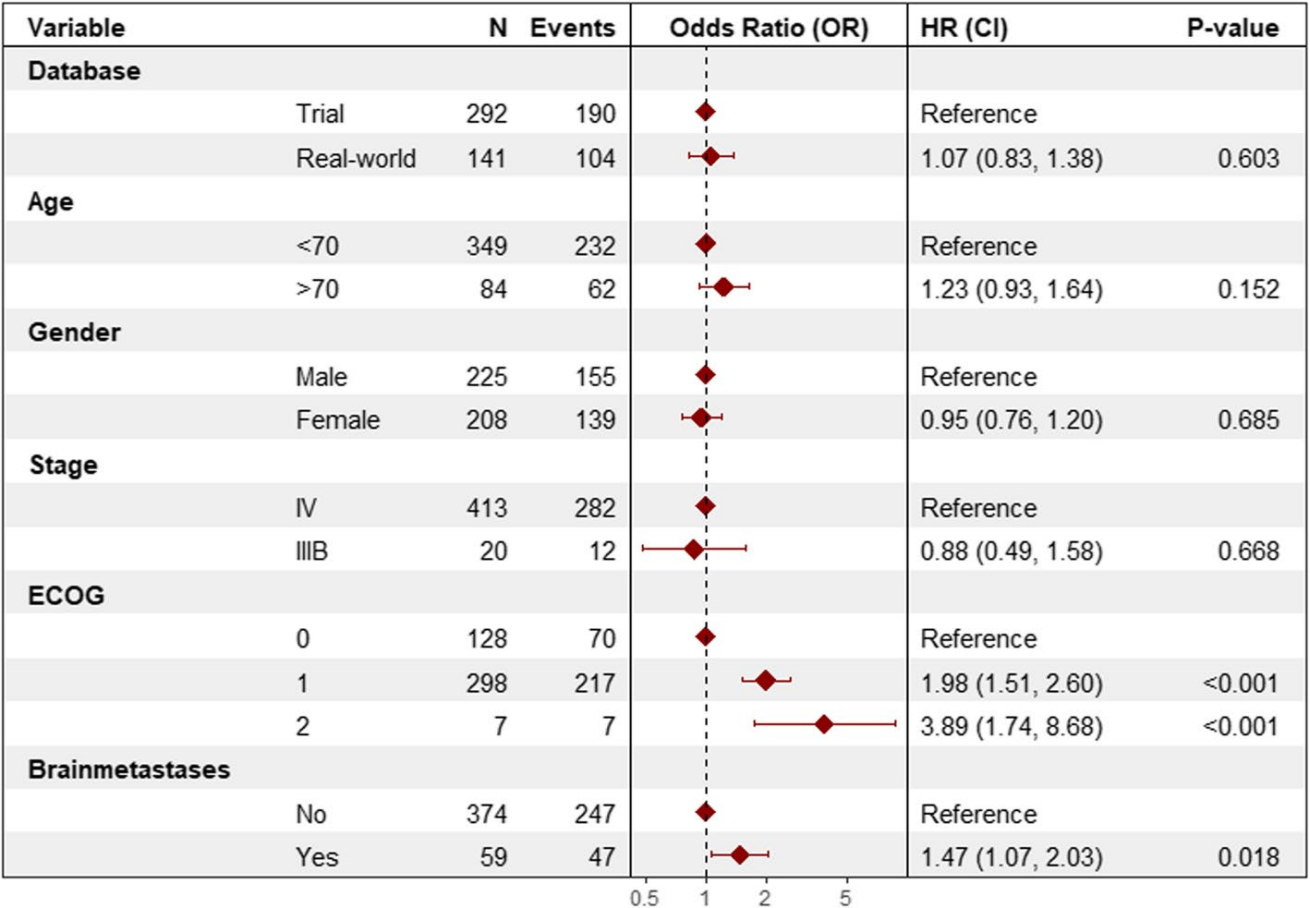
visualizing multivariate proportional hazard cox regression model of factors associated with overall survival (OS) of metastatic NSCLC patients

The median OS was 8.25 (95%CI: 6.93-13.2) months for real-world patients and 12.2 (95%CI: 9.90-15.1) months for trial patients (*p* = 0.33) (Fig. 3). ECOG PS 1 (*p* < 0.001) and ECOG PS 2 (*p* = 0.001), and PD-L1 expression > 50% (p = 0.001) were significantly associated with OS (Table 3). The unadjusted and fully adjusted HR for real-world versus trials were 1.13 (95%CI: 0.88-1.44) and 1.07 (95%CI: 0.83-1.38), respectively (Fig. 4).

**Table 3:** Univariate analysis (OS) of the pooled dataset including real-world and clinical trial patients

Variable	n	OR (95%CI)	P-value
Population			
Trial patients	292	Reference	
Real-world patients	141	1.13 (0.88-1.44)	0.34
Age (years)			
< 70	349	Reference	
≥ 70	84	1.25 (0.94-1.66)	0.118
Gender			
Male	225	Reference	
Female	208	0.92 (0.73-1.15)	0.454
Stage			
IV	413	Reference	
IIIB	20	0.85 (0.48-1.22)	0.584
ECOG PS			
0	128	Reference	
1	298	1.95 (1.48-2.55)	< 0.001
2	7	3.82 (1.75-8.33)	0.001
Presence of brain metastases			
No	374	Reference	
Yes	59	1.35 (0.99-1.85)	0.059
Histology tumor			
Adenocarcinoma	400	Reference	
Large cell carcinoma	15	1.24 (0.68-2.26)	0.488
Adenosquamous	3	0.94 (0.23-3.79)	0.932
Other	11	0.59 (0.24-1.43)	0.246
Not otherwise specified (NOS)	3	2.45 (0.78-7.65)	0.123
PD-L1 expression			
< 1%	141	Reference	
1-49%	86	0.92 (0.66-1.28)	0.606
> 50%	76	0.53 (0.36-0.77)	0.001
Unknown	130	1.24 (0.94-1.63)	0.13

*ECOG PS* Eastern Cooperative Oncology Group Performance Score, *PD-L1* Programmed death-ligand 1, *OR* Odds Ratio

CI = Confidence interval, ECOG = Eastern Cooperative Oncology Group Performance Score, HR = Hazard Ratio. Analysis from the pooled dataset including real-world and clinical trial patients.

## Discussion

This study with IPD from both real-world patients and trial participants showed that through the arisen possibility of multivariable modeling potential causative factors for an efficacy-effectiveness gap can be identified. For OS, the HR for real-world versus trials moved to 1.07 (0.83-1.83) after adjustment, suggesting that differences in the available characteristics between the two settings partly explain the altered OS seen in real-world practice. The latter phenomenon was not observed for PFS, suggesting that for that outcome other unmeasured factors are involved.

The median PFS of real-world patients was longer compared to trial patients, resulting in an HR for PFS below 1.00. Although ECOG PS was statistically significant in the multivariate Cox analyses, the adjusted HR between real-world and trial patients did not change. The etiology for this gap in PFS is believed to be multifactorial, with contributing factors including differences in patient populations, healthcare delivery, and variability in the experience of treating health care providers. Multiple factors which could explain differences in patient populations were measured but did not lead to a difference in HR. Unmeasured factors involving PFS could be smoking status, comorbidities, and frailty. Previous research also showed that use of corticosteroids and the number of organs with metastases are associated with PFS [14]. Healthcare delivery was different in terms of response measurement. According to the original Checkmate-057 trial study protocol, response was evaluated in week 9 after nivolumab initiation and every 6 weeks thereafter [15]. In real-world practice, response was assessed every 8 weeks. This led to visible drops in the Kaplan-Meier for PFS of trial patients, while these are less obvious in the real-world PFS **(**supplement 1**).** Furthermore, measuring progressive disease using the Response Evaluation Criteria in Solid Tumors (RECIST)- criteria can be less structured and strict in real-world than in trial patients [16]. In clinical practice, the immune responses assigned using RECIST (iRECIST) criteria are used, which include unconfirmed progression [17]. Consequently, conclusions about progressive disease might be delayed in clinical practice what could result in considering possibilities for subsequent systemic treatment later as well. Hypothetically, real-world patients remain treated with nivolumab while with progressive disease, in turn leading to further clinical deterioration reducing the tolerability of subsequent docetaxel, eventually leading to the inverse of the HR for overall survival.

In contrast to PFS, the non-significant difference in OS between real-world and trial shifted towards a null effect after adjustment for the available characteristics in the data (aHR of 1.07 (95%CI, 0.83-1.38)). This suggests that differences in ECOG PS and presence of brain metastases are linked to the observed shorter OS in real-world practice.

Apart from the beforementioned potential, this study also confirms the results using the standard approach of trial and real-world comparison using software applications. The unadjusted calculated HRs for PFS and OS in the study of Cramer-van der Welle et al are identical to the findings of this study using IPD [10].

A strength of our study we consider the quality of the real-world data. Data were manually extracted from electronic healthcare records and with very few missing data. An exception is the PD-L1 expression status which was often missing in real-world (48.9%) since it is not mandatory to measure this before nivolumab treatment in second line. We therefore could not use this factor in the multivariate analyses. Besides this, we could also not test for smoking status that in the Checkmate-057 study was an effect modifier (less effect in never smokers). On the other hand, we expect most patients to be current or past smokers. Altogether, we argue that most of the characteristics with the high prognostic value were included in the analyses [4]. A possible limitation was that the trial data only included PFS and OS calculated from the date of randomization and not from the start of nivolumab treatment as in real-world practice. However, as stated in the RCT protocol, nivolumab treatment should be initiated within three business days after randomization [15]. This very short period is unlikely to affect the outcomes of this study and will not introduce bias in the comparison with the Cramer et al. paper because that study calculated survival times similarly. Finally, we focused in this study on the relative changes in the HR and not on significancy. In case only aggregated trial data are available, a covariate balancing method analogous to propensity score weighting could be used [18].

In the present study we assessed the value of IPD with second line nivolumab, while Cramer-van der Welle et al. also reported a significant impaired OS in real-world with first line pembrolizumab. Unfortunately, due to unavailability of trial IPD on pembrolizumab, we could not assess what the added value of adjustment with IPD would be for that regimen. The European Medicines Agency (EMA) started an initiative to publish clinical trial data submitted to EMA as part of marketing authorization applications [19]. At the moment, trial data on COVID-19 medicines do become publicly available [20]. Hopefully, initiatives from the EMA and others like ClinicalStudyDataRequest.com will help to improve the availability of much more clinical trial data, considering the privacy of patients included in the trial, to allow better identification of factors associated with an efficacy-effectiveness gap (if any), in turn facilitating individualized prognoses and treatment planning [21–23].

## Conclusion

This study is an example how IPD from both real-world and trial patients can be applied to search for factors that could explain an efficacy-effectiveness gap. Making IPD from clinical trials available to the international research community allows this.

## Supplementary Information

**Additional file 1.**

## Data Availability

The datasets used and/or analysed during the current study are available from the corresponding author on reasonable request.

## Abbreviations

ECOG PS: Eastern Cooperative Oncology Group Performance Score
EE: Efficacy-effectiveness
EMA: European Medicines Agency
HR: Hazard Ratio
IPD: Individual patient data
NSCLC: Non-small cell lung cancer
OS: Overall survival
PD-L1: Programmed death-ligand 1
PFS: Progression-free survival
RCT: Randomized controlled trial
RECIST: Response Evaluation Criteria in Solid Tumors
