# Correction: Individual patient data to allow a more elaborated comparison of trial results with real-world outcomes from second-line immunotherapy in NSCLC

**DOI:** 10.1186/s12874-023-01850-7

**Published:** 2023-01-27

**Authors:** R. K. Ismail, F. M. N. H. Schramel, M. van Dartel, A. M. G. Pasmooij, C. M. Cramer-van der Welle, D. L. Hilarius, A. de Boer, M. W. J. M. Wouters, E. M. W. van de Garde

**Affiliations:** 1 Dutch Institute for Clinical Auditing, Leiden, the Netherlands; 2 Division of Pharmacoepidemiology and Clinical Pharmacology, Utrecht Institute for Pharmaceutical Sciences, Utrecht, The Netherlands; 3 Medicines Evaluation Board, Utrecht, The Netherlands; 4 Department of Pulmonary Diseases, St Antonius Hospital, Nieuwegein, The Netherlands; 5 Santeon Hospital Group, Utrecht, The Netherlands; 6 Department of Clinical Pharmacy, Rode Kruis Hospital, Beverwijk, The Netherlands; 7 Department of Biomedical Data Sciences, Leiden University Medical Center, Leiden, The Netherlands; 8 Department of Clinical Pharmacy, St Antonius Hospital, Nieuwegein, Utrecht, The Netherlands

**Correction: BMC Medical Research Methodology 23, 1 (2023)**

**https://doi.org/10.1186/s12874-022-01760-0**

Following publication of the original article [1], the authors requested to correct the word “first-line” to “second-line” in the article title.

The original article has been updated.
