# Supplementary material for: Individual patient data to allow a more elaborated comparison of trial results with real-world outcomes from first-line immunotherapy in NSCLC
Source: BMC Med Res Methodol. 2023 Jan 3;23:1. doi: 10.1186/s12874-022-01760-0 (PMC9807419; doi:10.1186/s12874-022-01760-0)
Supplement: Supplementary file 1 — Additional file 1. [file 12874_2022_1760_MOESM1_ESM.docx]

# Supplement

## Supplement 1: Kaplan-Meier estimate of the PFS of real-world (a) and trial (b) metastatic NSCLC patients treated with nivolumab.


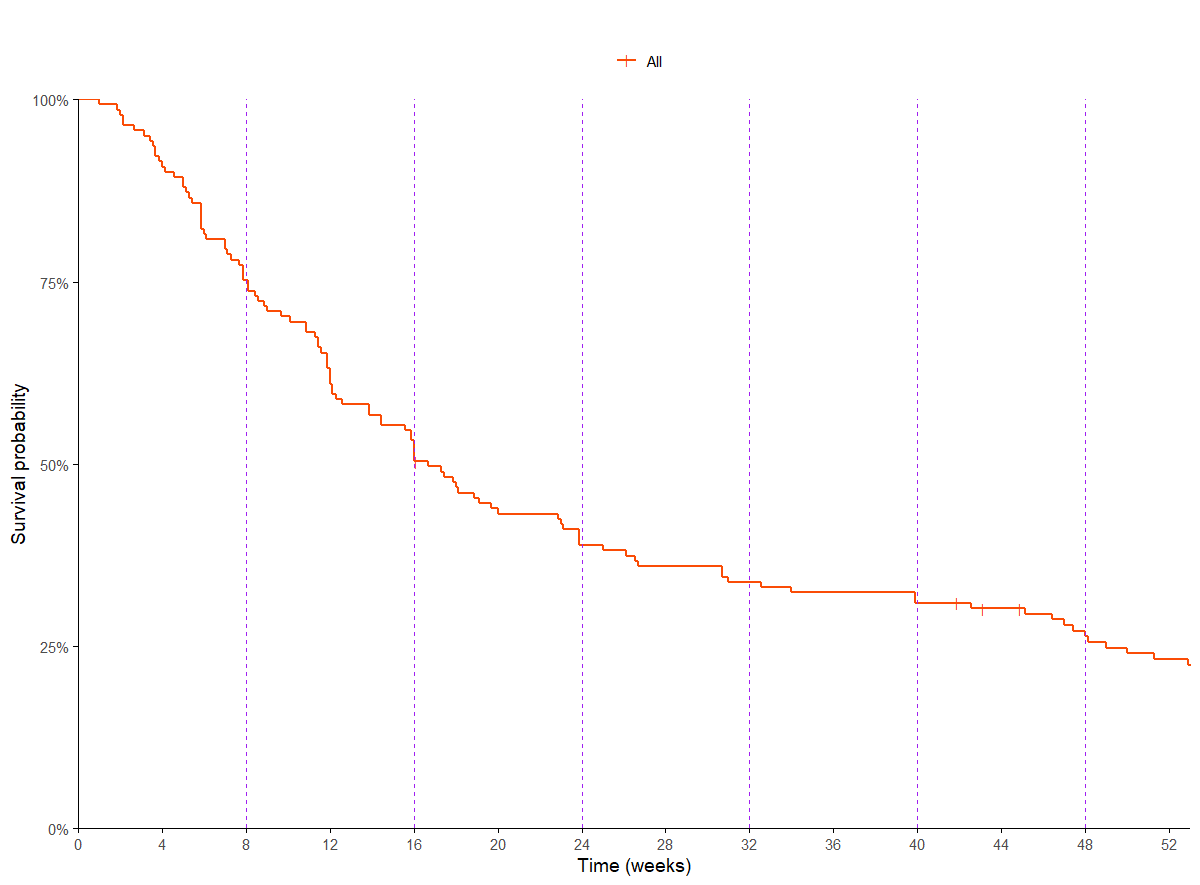


1. *Real-world patients in which response is evaluated every eight weeks in the first year of nivolumab treatment.*


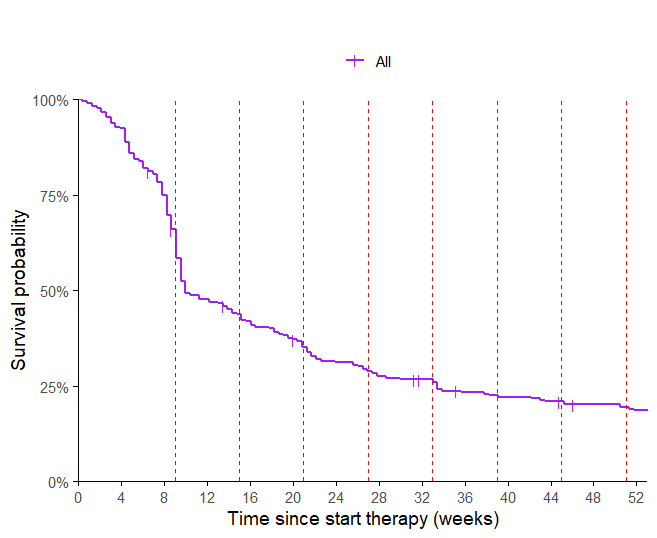


1. *Trial patients in which response was evaluated in week 9 after nivolumab initiation and then every six weeks from week 9, in the first year of nivolumab treatment.*
